# Evaluation of the free, open source software WordPress as electronic portfolio system in undergraduate medical education

**DOI:** 10.1186/s12909-016-0678-1

**Published:** 2016-06-03

**Authors:** Javier Avila, Kai Sostmann, Jan Breckwoldt, Harm Peters

**Affiliations:** Dieter Scheffner Center for Medical Education and Educational Research, Charité –Universitätsmedizin Berlin, Charitéplatz 1, Berlin, 10098 Germany

**Keywords:** ePortfolio, WordPress, Medical education, Software, Electronic portfolio

## Abstract

**Background:**

Electronic portfolios (ePortfolios) are used to document and support learning activities. E-portfolios with mobile capabilities allow even more flexibility. However, the development or acquisition of ePortfolio software is often costly, and at the same time, commercially available systems may not sufficiently fit the institution’s needs. The aim of this study was to design and evaluate an ePortfolio system with mobile capabilities using a commercially free and open source software solution.

**Methods:**

We created an online ePortfolio environment using the blogging software WordPress based on reported capability features of such software by a qualitative weight and sum method. Technical implementation and usability were evaluated by 25 medical students during their clinical training by quantitative and qualitative means using online questionnaires and focus groups.

**Results:**

The WordPress ePortfolio environment allowed students a broad spectrum of activities – often documented via mobile devices – like collection of multimedia evidences, posting reflections, messaging, web publishing, ePortfolio searches, collaborative learning, knowledge management in a content management system including a wiki and RSS feeds, and the use of aid tools for studying. The students’ experience with WordPress revealed a few technical problems, and this report provides workarounds. The WordPress ePortfolio was rated positively by the students as a content management system (67 % of the students), for exchange with other students (74 %), as a note pad for reflections (53 %) and for its potential as an information source for assessment (48 %) and exchange with a mentor (68 %). On the negative side, 74 % of the students in this pilot study did not find it easy to get started with the system, and 63 % rated the ePortfolio as not being user-friendly. Qualitative analysis indicated a need for more introductory information and training.

**Conclusions:**

It is possible to build an advanced ePortfolio system with mobile capabilities with the free and open source software WordPress. This allows institutions without proprietary software to build a sophisticated ePortfolio system adapted to their needs with relatively few resources. The implementation of WordPress should be accompanied by introductory courses in the use of the software and its apps in order to facilitate its usability.

## Background

The use of logbooks to track students’ or trainees’ learning activities is increasingly common in medical education [1]. Ideally, portfolios include additional features that stimulate acquisition of knowledge, practical skills and professional development. As an example, portfolios may contain critical reflection on learning, thereby challenging the learner’s performance in a way that logbooks do not do [2]. Electronic portfolios (ePortfolios) allow for features such as providing feedback on the learning outcome and progress [3]. However, evidence supporting the usefulness of ePortfolios in the undergraduate education is limited [1].

Much of the learning evidence in medical education comes from practical training activities in hospital wards and outpatient clinics. In this setting, an ePortfolio system with mobile capabilities would add extra value by providing the opportunity to collect and store evidence on learning during clinical training without the need of a desktop or laptop computer. Nowadays, most medical students own technically advanced handheld digital devices with multimedia, i.e. text, pictures, audio or video capabilities [4]. Even if an ePortfolio system may not be fully operative from mobile devices. These devices are able to capture multimedia or record event reminders and send the data remotely to the ePortfolio and attach them to the entries carried out on a desktop computer [5].

E-portfolios are often implemented technically using in-house developed software tailored to the needs of the institution [6, 7]. However, developing such an IT solution, which ideally should also include mobile capabilities for all different types of devices, is resource-intensive. A second approach could involve the use of external, commercially available ePortfolio software with mobile capabilities. However, this is expensive as well and more difficult to adjust to the specific requirements of the institution. A third opportunity for acquiring an ePortfolio, which has recently emerged, may be the use of free and open source Web 2.0 solutions. This kind of software is less resource-intensive, allows for adjustments and has already been successfully implemented for building low-cost platforms for e-learning activities [8].

WordPress is a free open source blogging tool and content management system, and runs on a web hosting service that potentially may be used as an ePortfolio system. It is the most popular blogging system on the web [9] with more than 60 million websites [10]. The software can be downloaded to run and host one's own service. The popularity and wide-spread use of this software confers it with powerful capabilities that can be added as plugins and extend many of its functionalities including the ones needed for an ePortfolio. Most of these plugins can be downloaded and custom installed free of charge. Especially together with knowledge of the PHP programming language, small adjustments can also be made with relatively little time investment.

The purpose of this study was to create an ePortfolio system with mobile capabilities for undergraduate medical education using WordPress. The system was evaluated from a technical perspective and by a group of undergraduate medical students using quantitative and qualitative methods via an online questionnaire and focus groups. The primary goal of this study was to set up a sophisticated WordPress ePortfolio platform with multiple capabilities, including mobile options, and to test it from a technical und usability point of view. The WordPress ePortfolio served as an add-on to students obligatory, summative curricular activities and assessments and involved a facultative and formative approach to students' learning. To our knowledge, this is the first report evaluating this blogging Web 2.0 tool as an ePortfolio.

## Methods

### Technical platform

The freely available WordPress version 3.4.2 was downloaded, installed and configured as a multisite environment on a Microsoft Windows server 2008 R2 running a XAMPP web server (version 1.8.0). In order to test for additional functionalities, we installed a battery of 43 plugins that could be useful for medical students, and made small adjustments with the help of some PHP code (see Tables 1 and 2). Key criteria to evaluate an ePortfolio system have been previously established [11–13]. Our WordPress ePortfolio system met most of these criteria (see Table 3).

**Table 1:** Basic functions of the WordPress ePortfolio

Functionality	Built-in or Plugin required
Annotations and reflections, online text editing	Built-in
Internal links and external links	Built-in
File upload	Built-in
Configuration of templates	Plugin
Comments	Built-in
Comment rating	Plugin
Configuration of built-in templates	Built-in
Detailed configuration of access rights	Plugin
Assessment and presentation	Built-in
Metadata (configurable tags, types and categories)	Built-in enhanced with plugins
Web publishing	Built-in
Syndication	Built-in enhanced with plugins
Internal and external notifications/messaging	Plugin
Search of artifacts	Built-in enhanced with plugins
Configuration of a file/document management centre	Built-in enhanced with plugins
Export and import of the ePortfolio that allow one to continue using the ePortfolio after leaving the university or coming from another university	Built-in
Mobile access (from iPads, iPhones, android tablets and android phones)	Built-in
Tracking of user activity	Not implemented but possible with plugins
Configuration of language	Built-in

**Table 2:** Additional functions included in the WordPress ePortfolio

Functionality	Built-in or Plugin required
Administration: addition of multiple users	Plugin
To do list	Plugin
Form builder	Plugin
Progress bar for projects or works	Plugin
Google doc embedder	Plugin
HTML5 and Flash Video Player	Plugin
iframe embedder for YouTube	Plugin
Quiz builder	Plugin
Configuration of Dashboard metaboxes	Plugin
Table creator	Plugin

**Table 3:** Criteria checklist for the evaluation of ePortfolio Software from Himpsl and Baumgarten [13]

	Availability
Essential criteria	
Input of keywords	Yes
Internal cross-references	Yes
External cross-references	Yes
Publication in the web	Yes
Pricing and license schemes	Not applicable
Simple data export	Yes
Support of all currently used A-grade browsers	Yes
Collecting, organizing, selecting	
Simple data import	Yes
Comfortable data import	Yes
Searching, sequencing and filtering	Yes
Annotations to files	Yes
Aggregating (integration of external data via feeds)	Yes
Version control of files	Yes
Reflecting, testing, verifying and planning	
Guidelines for reflection	No, but could be built-in
Guidelines for competences	No, but could be built-in
Guidelines for evaluation (self-assessment, assessment by others)	No, but could be built-in
Guidelines for goals, personal development and career management	No, but could be built-in
Guidelines for Feedback (advice, tutoring, mentoring)	No, but could be built-in
Representing and publishing	
Access control by users (owner, peers, authority, public)	Yes
Adaptation of the display: layout (flexible placing, boilerplates)	Yes
Adaptation of the display: colours, fonts, design	Yes
Publishing of several portfolios, or alternatively, various views	No
Administrating, implementing, adapting	
Development potential of the provider, company profile	Yes
Enabling technologies (programming language, operating system, …)	Yes
Authentication and user administration (backed-up interfaces, …)	Yes
E-Learning-standards	Yes
Migration/storage/export	Yes
Usability	
User interface	Yes
Syndicating (choice of feeds for the individual portfolio)	Yes
Availability, accessibility	Yes
Navigation/initial training/help	Online help
External and internal information function	No
Interchangeable, adaptable user-defined boilerplates	No, but could be built-in
Personal storage, respectively export function	Yes

**Fig. 1:**
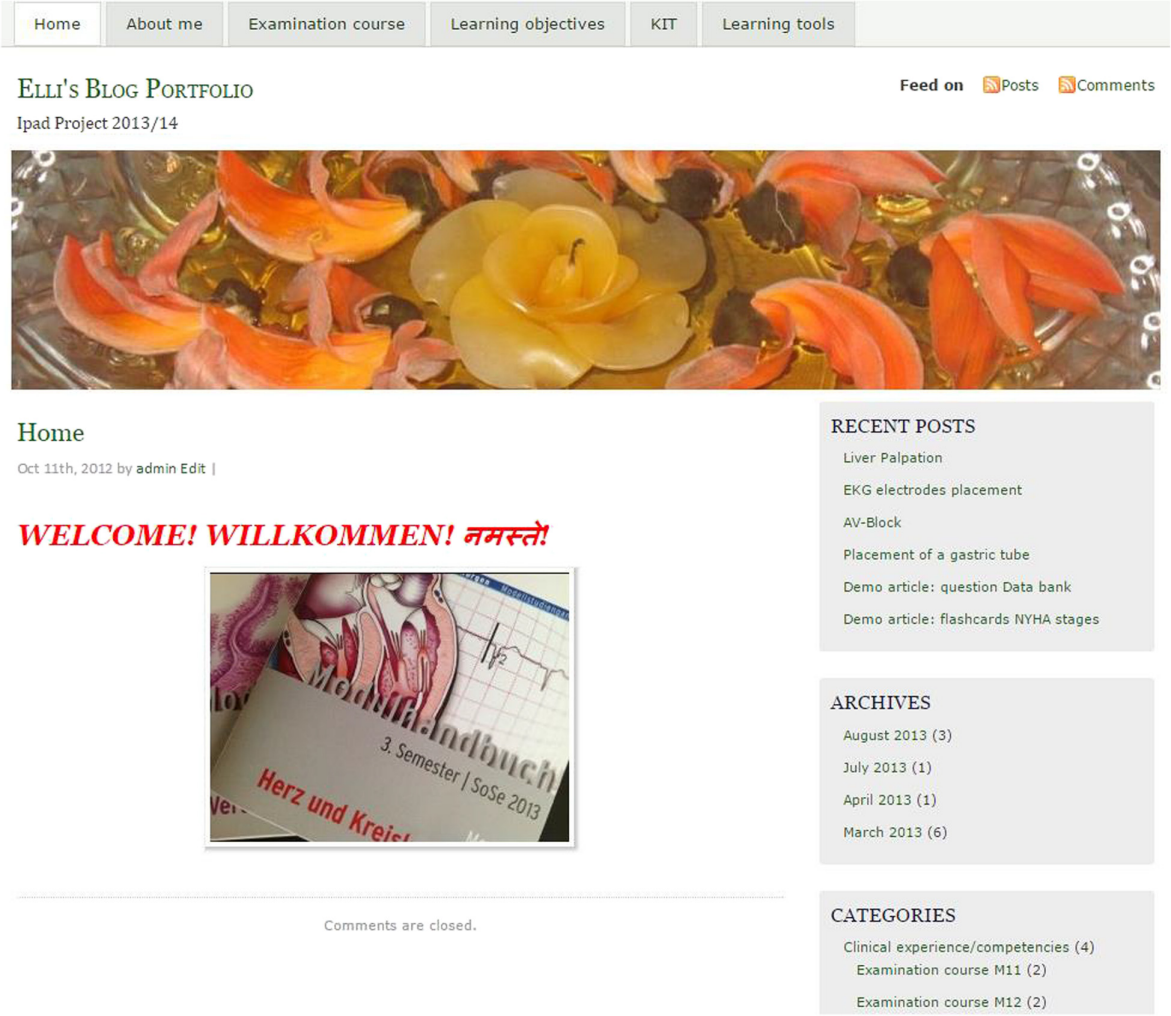
Example of a personalized layout of the WordPress ePortfolio

Once the system was operating, we created a standard starter template for the ePortfolio, which our students could modify according to their needs. This template included menu items for self-introduction of the student and his or her interests as well as for entering evidence and reflections regarding the learning content and objectives. It also included tools for knowledge management. Students were able to change the starter template in various ways and they were free to create and upload their own contents. Students could change any appearance features of the ePortfolio such as background colour, fonts and general layout very easily and quickly using the templates provided. The possibility of allowing students to personalize their own ePortfolio is regarded as an important feature of an ePortfolio (see Table 3) [13]. Administration and all functions of the ePortfolio remain unchanged and cannot by altered by the students. Figure 1 shows an example of the WordPress ePortfolio layout. Except for minor details, the initially established ePortfolio platform was not changed once the study had started.

The ePortfolio system was password protected. Each student was the administrator of his own ePortfolio and could assign access rights to other students. In addition, our technical research staff had access to all ePortfolios and could thereby follow and analyse their activities.

### Students’ acquisition and implementation of the system

We recruited third-semester medical students for the study with the help of flyers and emails. Their participation was voluntary and candidates were chosen in the order of their application. Participating students had to sign an informed consent before getting access to the ePortfolio system. In addition, they had to confirm that they would not publish any data that could be used to identify patients.

A group of 25 third-semester medical students was enrolled to test the WordPress-based ePortfolio system. The study took place during the summer term 2013, when the integrated modules “Cardiovascular System” and “Digestive System and Metabolism” were held. Part of these modules was a weekly clinical skills course on hospital wards with small group teaching for 6–8 students. Instruction was given by a physician, and in addition, students interviewed and examined patients on their own. Examples of learning objectives for the course were “conducting a medical interview and a physical examination focused on the cardiovascular (or abdominal) system”, “recording an electrocardiogram”, or “insertion of a nasogastric tube”. The clinical skills courses were not specifically modified to promote the use of an ePortfolio. There was no assignment to an ePortfolio mentor.

Students were provided with an iPad (20 units, leased by Charité) or an android Samsung Galaxy Note 10.1 tablet (5 units, provided by Samsung Electronics GmbH). At the beginning of the study, the students received a 90 minute face-to-face introduction to ePortfolios and the basic technical aspects of using WordPress. We put special emphasis on using the ePortfolio from the provided mobile devices, also from smartphones, to specifically test the mobile capabilities of the WordPress-based ePortfolio. Each student built his/her individual ePortfolio during the course of the semester. The main assignments were to collect evidence during clinical training time on achievement of the learning objectives, and to reflect on the learning process and progress.

### Quantitative and qualitative analysis

At the end of the semester, we evaluated the students’ experiences via an online questionnaire. The questionnaire included 21 questions on demographic data, computer literacy and the usability of the ePortfolio. Most items were to be rated on 5-point Likert scales (from “fully applies” to “fully not applies”), whilst for some questions free text could be entered. Students’ demographic data were expressed as mean plus/minus standard deviation. WordPress technical implementation and usability was analysed by descriptive qualitative statistics. In addition, two 90-minute long, moderated focus group sessions with either 5 or 15 students were carried out. The sessions were recorded and transcribed. The transcripts were analysed for main topics arising in the discussion and were categorized in a qualitative manner.

## Results

### Technical set-up and realization

From a technical point of view, WordPress allowed us to build a multimedia enriched ePortfolio system by expanding its basic functions with additional and freely available plugins. This was possible due to the high flexibility and great variety of plugins provided by WordPress, in combination with simple PHP programming. The resulting ePortfolio contained tools for capturing and collecting multimedia evidences, posting reflections, web publishing, search functions, collaborative learning, messaging system, knowledge management in a content management system including wiki and RSS feeds, online medical knowledge databases search center, and aid tools for studying (e.g. flashcards or self-assessment quizzes). Table 1 gives an overview of all basic functionalities and the extra features we added for test purposes. Table 4 shows an overview of what the students in our study were able to accomplish with the provided system. Figure 1 demonstrates a screen shot of a personalized layout.

**Table 4:** User capabilities in the WordPress ePortfolio

Capability (the ePortfolio allows the user to…)	Comment
Record experiences and reflections and collect evidences related to training*	This can be done with any kind of computer or mobile device with access to the internet. All these entries can be enriched with multimedia material (embedded pictures, audio, video, documents or mind maps)
Share contents with other students or subscribers of the ePortfolio	The subscribers have to be granted access rights by the user first, in order to have access to the ePortfolio
Comment and discuss postings*	This can be done in a similar way to that of usual social platforms (also including comment and post rating or like functions).
Use the ePortfolio as Content Management System and therefore as Knowledge Management System	The ePortfolio is equipped with file uploading, search, filtering and metadata tagging functions that are very convenient for this purpose
Use the ePortfolio as a tool for collaborative learning and research	We embedded the Wiki system of our institution and search text fields for the main medical knowledge databases (like UpToDate, PubMed, etc.) for this purpose
Use the ePortfolio as a learning aid	Plugins allow the students to design quizzes and create flashcards
Exchange with other ePortfolios through broadcasting/feeds to/from other ePortfolios	Posts can be published in several ePortfolios simultaneously and update tickers about new posts from other ePortfolios can be embedded. Access rights have to be provided first
Personalize the ePortfolio	The layout and content (also Menus, widgets, etc.) can be changed. Many layout templates are available.

* Indicates that the feature is available on the mobile app

**Fig. 2:**
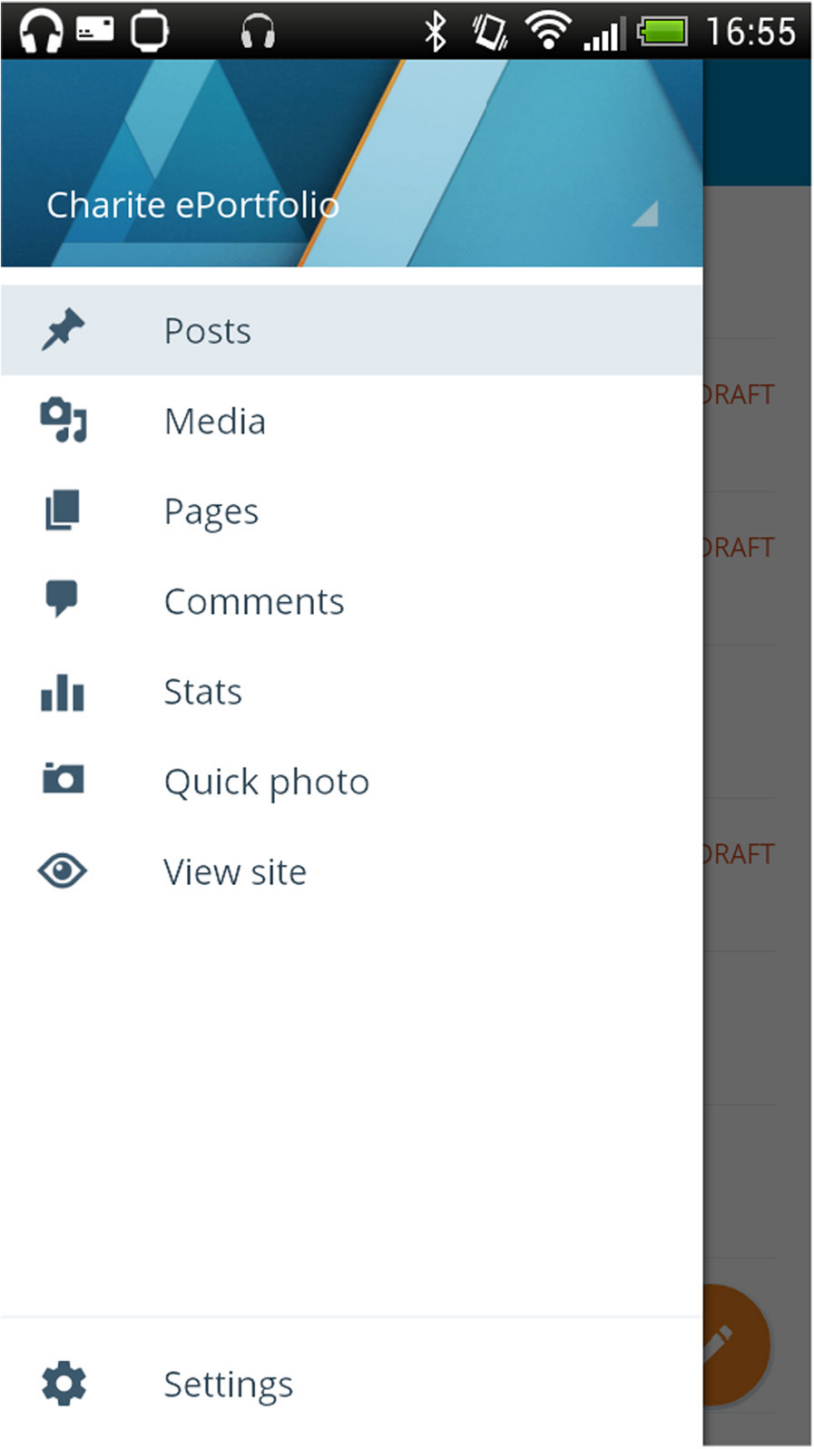
General screen view of the WordPress ePortfolio app on a mobile device

Another important feature of the WordPress ePortfolio was its mobile capabilities. The WordPress app allowed the users to send posts directly to the ePortfolio and then capture and upload multimedia, tag artifacts and write reflections during their clinical training without the need of a computer. It was possible to save these data while being offline and send them to the ePortfolio later. Some students used only the app to build their ePortfolio, not making use of computers at any moment. Figure 2 depicts a screen shot of the general appearance of the WordPress ePortfolio app on a mobile device.

The set-up of the WordPress-based ePortfolio system needed relatively few resources. The installation and configuration required about 20 hours of work and no specialized skills in computer science. The requirements on the hardware are dependent on the number of students’ ePortfolios to be hosted. We used a Microsoft Windows server 2008 R machine with a 2.27 GHz Intel Xeon CPU, 2 GB of RAM memory and a 50 GB Hard drive for our study.

### Students’ characteristics and general use of the WordPress-based ePortfolio

None of the students in our study had ever used an ePortfolio before. Out of the 25 students who started in the study, two finished the project after one month, because they did not have enough time to build the ePortfolio; one of these (21 years old) used an iPad, and the other one (also 21) a Samsung tablet. Of the remaining 23 students, 19 completed the online questionnaire (response rate: 82 %). The mean age of these 23 participants was 23 ± 5.5, 57 % were females. 95 % had experience using computers, email and internet, and 78 % owned a smartphone. 52 % had used learning software before and 48 % virtual networks. The fraction of pre-study use of podcasts was 26 %, of Wiki 22 % and of forums 30 %.

All students used the ePortfolio and filled out the templates provided. Over and above that, approximately 50 % of the students built personalized and visually appealing ePortfolios (see Fig. 1), which necessitated a change of the layout according to their own personal needs and their entering of at least two posts. The use of WordPress as an ePortfolio environment allowed for dynamic and rich ePortfolios through the use of pictures, videos, quizzes and discussion threads that showed the potential for an interaction between faculty and students, and among the students themselves. The students’ posts on clinical experiences were commented and rated by peers in a similar way as in other social platforms through “like buttons” that enabled comments popularity lists.

Over the eight weeks of study duration, students uploaded 53 text-only posts, 48 comments, 23 videos with or without text and 60 pictures with or without text. Most text posts had 5–20 lines, and some were even several pages long. The students posted reflexions, links to learning resources, summaries of learning objectives, clinical cases and quizzed their peers on different topics or cases. Most of the content was self-created. Most artifacts were captured during seminars and practical training likely by use of mobile devices; 13 YouTube video embeddings were uploaded. Students interacted with each other through comments, including questions, answers, praise or suggestions of 1 to 9 lines length. In the eight-week study period, students spent on the average 10 hours publishing posts, managing and configuring their ePortfolio. Students mainly utilized basic WordPress features like posting text, pictures or videos and comments. The plugin most frequently used was the function to embed YouTube videos.

During the implementation phase, students experienced and reported technical problems when using WordPress on mobile devices. First, on iPads it was not possible to play videos. We found out that installation of the mod_xsendfile module was necessary to be able to play videos on a multisite blogging system on iPads. It was not possible to install this module on our Windows server 8 R2, but it should be possible to install this on a Linux system. This had been shown by other users of WordPress on multisite environments. Another, albeit more inconvenient workaround for this problem, was the changing of the paths of the videos every time they had to be uploaded. For this reason, we recommend using WordPress multisite on a Linux server.

Secondly, there were problems with embedding YouTube videos or uploading videos from mobile devices (iPads or android tablets). As a workaround, we had to turn on the ‘script language use’ option for users, which may represent a security risk on a multisite installation. This problem can be solved only in part with the installation of plugins to allow for the functions without the use of script language. To guarantee high security of the multisite network it might be necessary to turn some functions off, thereby disabling/reducing especially some of the mobile capabilities of the system.

### Quantitative evaluation of the WordPress based ePortfolio

**Fig. 3:**
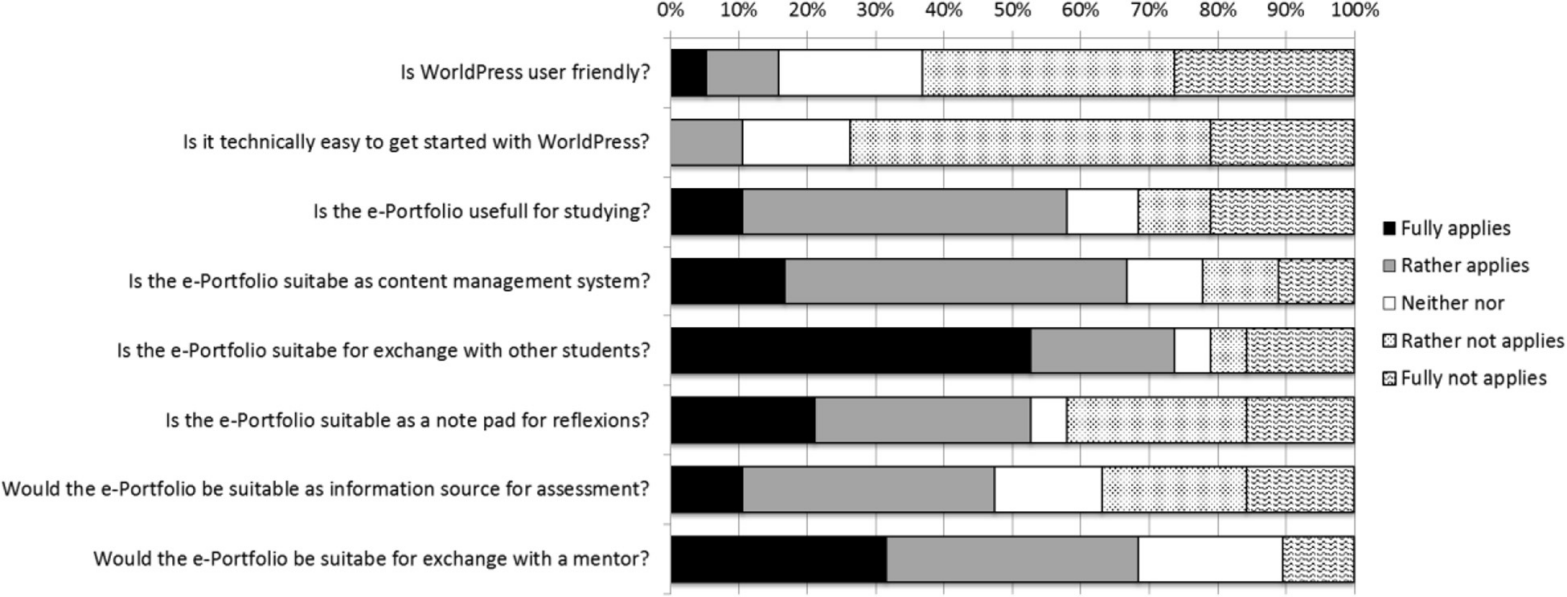
Results on the students' rating of the usability and technical capabilities of the created WordPress-based ePortfolio system

Main results from the online questionnaire are shown in Fig. 3. Students rated positively (fully and partly agree) the usefulness of the WordPress ePortfolio system for studying (58 %) as well as its suitability as a content management system (67 %), for exchange with other students (74 %) and as a note pad for reflections (53 %). Without having tested these capabilities in practice, the students fully and partly agreed on the suitability of the WordPress ePortfolio system as a tool to assess their learning progress (48 %) and to exchange with a mentor (68 %).

In terms of the usability of the system, only 16 % of the students found the WordPress ePortfolio system user-friendly, while 63 % gave negative ratings (“rather not” or “not at all”). Eleven per cent of the students found it easy to get started with the WordPress-based ePortfolio while 74 % did not find it easy. Seventy-one percent of the students stated that the option to change the WordPress template layout had no impact on its usability, while 21 % thought that it did improve the usability. In contrast, 46 % of the students thought that the possibility to change the appearance of the WordPress ePortfolio encouraged them to use it more often and find it more useful, while 15 % said that that didn´t matter. Sixty-two percent of the students noted that the mobile capabilities of the WordPress ePortfolio made it easier to publish artifacts.

### Qualitative evaluation

Online narrative comments on the use of the WordPress-based ePortfolio system were confirmed by the focus groups’ interviews and analysis. In the following, we summarize the topics discussed and quote specific statements for illustration. Students stated that the ePortfolio was very useful for exchange with other students. The system allowed them to make and rate comments and to attach these comments to posts published by the owner of the ePortfolio. Any reflection or learning experience with or without multimedia capture could be shared with peers subscribed to the ePortfolio. This motivated students to discuss topics and publish posts *(“I was curious to know what other students publish”, “Great for networking, to communicate and discuss topics with others“).*

Many students thought that the ePortfolio would be a good way for a mentor to supervise and assess their progress and learning achievements. However, they pointed out that online supervision would have to be complemented by meeting them in person and it should not be the only mode of communication *(“My mentor should know me personally”, “It would be great to have a doctor as a mentor from the beginning of my studies supervising me through the ePortfolio”).*

There were several reasons for not being able to work fluently with the WordPress-based ePortfolio system especially at the beginning: most students were used neither to blogging software nor to an ePortfolio. Due to its many functions, WordPress required training for them to know how to use the program. At the beginning, students had to invest a considerable amount of time and effort to get acquainted with the system. After this period, students worked successfully with the blogging system. *(“The use of the WordPress app wasn’t easy at first but then it worked”, “The software had too many options”, “It wasn’t so easy to understand the functionalities of the software at first”, “I don’t want to have to configure so many things”, “WordPress was not as intuitive as Facebook”*). As to the solutions for these problems, the students found that more meetings and training would help *(“We would need more meetings at the beginning”*).

A few students also experienced technical problems with the iPad WordPress app especially when uploading media files. *(“The app crashed constantly”, “The app never completed the file upload”).* For many of the students in our study group, the app did not have an intuitive interface.

## Discussion

The provision of a technically advanced ePortfolio with a reasonable resource investment is a challenge. The present study shows that 1) the free open source software WordPress allows to build a technically advanced ePortfolio system according to reported standard references, and 2) in an implementation study, undergraduate medical students used this ePortfolio successfully alongside their clinical learning. In the following, we now discuss the basis and the relevance of these findings in detail.

The functionalities and content of the WordPress-based ePortfolio were implemented following recently published reference criteria with six major areas ranging from “essential criteria”, “collecting, organizing, selecting”, “reflecting, testing, verifying and planning”, “representing and publishing”, “administering, implementing and adopting” to “usability” [13]. From this checklist of 34 criteria, only two functions were not implemented when creating the WordPress ePortfolio, i.e. “publishing of several portfolios, or alternatively, various views” and “external and internal information function”. To integrate most of these essential features, it was not necessary to install additional plugins. Thus, the open source software WordPress allows the introduction of an advanced and sophisticated ePortfolio, which can serve as a viable digital tool to create electronic portfolios for undergraduate medical students. In addition, WordPress allows for cross-platform use on computers, tablets and smartphones. That makes access from different locations to the ePortfolio easier. This pilot study confirmed the advanced mobile capabilities of WordPress. This integration of mobile devices in the ePortfolio through apps is another strong point of WordPress. The students could capture and upload multimedia contents as evidence material from mobile devices during real clinical learning on the hospital wards. Another advantage of WordPress is its flexibility concerning layout and personalization. Almost half of our students felt that this feature was a motivation to use the ePortfolio platform more often. From a technical point of view, the personalization of the WordPress ePortfolio did not interfere with its functions and administration.

The technical set-up of the WordPress ePortfolio is easy to manage. Equally important, the set-up required only little manpower time and hardware resource. The requirements on the hardware are dependent on the number of students’ ePortfolios to be hosted but also on other factors like the size of the blogs, the number of plugins installed and the network traffic. With a network traffic of only a few requests per minute, our server processing power could have theoretically handled a few thousands blogs.

The feature of WordPress as a free open source Web 2.0 software has many further advantages. Given the popularity of this blogging tool and that it uses the power of the widely used database MySQL and the server software Apache (both also open source) the lifelong capabilities of the WordPress ePortfolios are practically guaranteed. WordPress is powerful due to its large developer community. Thus, a wide variety of plugins and extensions allow for tailoring to fit nearly every ePortfolio need. A simpler version of the ePortfolio could even be hosted on the WordPress website for free not requiring any kind of installation. The import and export functions built into the software allow for use beyond the medical school program. These files use the XML standard and are therefore readable by a wide variety of software, and not only by WordPress itself.

Many other ePortfolio solutions are bounded to the institution with proprietary software and data management without export functions. Contents created in WordPress can be easily exported for later use on other systems, contributing thereby to the acquisition of lifelong learning competencies.

An important point in any computer system is data safety. WordPress allows for password-protected posts and sites. The popularity of WordPress increases the risk of hacking attempts. To protect the system from these, it is important to always keep the WordPress installation and its plugins updated to their latest version. For maximum security is also important to restrict the use of script language for users.

This implementation study of a WordPress-based ePortfolio system with third semester students went along relatively smoothly. A few minor technical problems were identified but it was always possible to find workarounds. The students especially valued the WordPress ePortfolio for content managing, exchange with other students and note taking of reflections. Although this was not tested in the present study, our students valued the potential of the WordPress ePortfolio for interaction with a mentor and for assessment.

The students´ preferences about WordPress functionalities are similar to the features they normally value on social media platforms. Rating each other’s postings as well as various collaborative functionalities were highly appreciated by the students. These can be used to work with a mentor and for collaboration with peers. WordPress allows for media embedding of any kind, which is also widely used on social media. These features could motivate students to publish more.

However, there are some drawbacks to be discussed. Students´ feedback revealed that they didn´t find it easy to start using the WordPress ePortfolio system and they expressed a need for more introductory course time. This led us to the conclusion that more time is required for introductory courses and that plugins should be strictly reduced to the necessary ones.

### Limitations of the study

We tested WordPress on a server environment with 25 personal sites only. Therefore, we cannot predict its stability and reliability for larger student cohorts. This was a single centre study within a newly established modular, competency-based medical curriculum. The results may not be directly extrapolated to other medical schools with different settings in their undergraduate curricula. The students taking part in this pilot study applied voluntarily to the project, which may represent a selection bias, especially regarding their technical skills

An ePortfolio represents the online underpinning of an education model promoting proactive lifelong learning supported by mentoring and the social component of interaction with peers. These important parts should be introduced together with the digital infrastructure. If that is not the case, no definite conclusions can be drawn regarding the students’ acceptance and benefit of the ePortfolio. Nonetheless, this was not the aim of our study. The primary aim of this study was to test the technical and usability aspects of WordPress including its mobile capabilities as the core for the technical infrastructure of an ePortfolio system. For this purpose, we deliberately employed a facultative and formative approach to students' learning and assessment offering the ePortfolio as an add-on to their regular and compulsory curricular activities. This study showed the technical suitability of WordPress as ePortfolio platform. More studies are needed to analyse its impact on facilitation of students' learning and progress. Building on a technically established WordPress ePortfolio platform, these educational studies could be, for instance, hypothesis-based and include student groups with and without ePortfolio support.

## Conclusions

The free and open source software WordPress provides a platform to build and implement a technically advanced ePortfolio system with mobile capabilities for undergraduate medical education. Institutions without proprietary software can use and adjust the WordPress ePortfolio system to meet their needs with relatively few resources. The implementation of a WordPress ePortfolio should be accompanied by sufficient introductory time for software and apps to facilitate its starting usability.

## Abbreviations

ePortfolio, electronic Portfolio
